# Editorial: Advances in biosulfidogenic processes

**DOI:** 10.3389/fbioe.2022.1025093

**Published:** 2022-10-26

**Authors:** Anna Patrícya Florentino, Javier Sánchez-España, Irene Sánchez-Andrea

**Affiliations:** ^1^ Laboratory of Microbiology, Wageningen University and Research, Wageningen, Netherlands; ^2^ Centro Nacional Instituto Geológico Minero de España (IGME), Madrid, Spain

**Keywords:** sulfur cycle, biosulfidogenesis, microbial sulfate reduction, bioremediation, metal removal, elemental sulfur recovery

The physiological adaptability of microorganisms thriving by respiration of sulfur compounds and generating sulfide as an end product makes them an attractive tool for biotechnological applications in a variety of settings. Such versatility entices the scientific community interest; and, therefore, engineering and microbiological techniques have been combined to design and improve biotechnological systems targeting biological sulfide production. The application of biosulfidogenesis has extensive possibilities, making it a noteworthy process for approaching environmental issues and reaping added-value products, therefore contributing to economical circularity.

Although the processes involved in biosulfidogenesis have long been understood, current developments in microbial physiology, last-generation molecular biology techniques that combine several “omics,” and engineering techniques have broadened the scope of this technology. This *Research Topic* issue comprises six original research articles, bringing forth a valuable update on biosulfidogenic processes with novel data on molecular, genomic, biochemical, physiological and applied aspects.

Three manuscripts focus their research on microbial sulfate reduction in acidic conditions, which has as target the remediation of acid mine drainage (AMD) and acid rock drainage (ARD)-impacted environments. Although biosulfidogenic microorganisms thriving at low pH have been isolated and characterized, they are commonly isolated from enrichments obtained from sediments of acidic environments. Ayala-Muñoz et al. focus their investigation on the overlooked water column of acidic environments and revealed such environments as a potential source of novel microorganisms responsible for complete conversions of sulfur compounds. The combination of metagenomic and metatranscriptomic analyses in an acidic and deeply stratified pit lake have allowed these authors to find novel taxa from *Actinobacteria, Chloroflexi* and *Nitrospirae* phyla which were abundant and show an important capability of biosulfidogenesis. The information reported by the authors provides multiple insights on how to improve sulfide production and consequently enhance specific communities/species of microorganisms.

Ilin et al. look at the geochemical and microbiological aspects of sulfate reduction in AMD environments to explain how biosulfidogenesis leads to metal sulfide formation and proton consumption even in very acidic environments. This combination of metal removal (through microbially induced formation of sulfide minerals like CuS or ZnS) and acidity consumption represents an efficient and cost-effective process that naturally attenuates the environmental impacts of AMD-affected areas. Their study shows that the amendment of these usually oligotrophic systems with a suitable organic electron donor like glycerol triggers biosulfidogenesis, and the increase in pH due to proton consumption allows for the enrichment and community establishment of moderately acidophilic and acidotolerant bacterial groups, such as *Desulfurispora, Desulfovibrio, Desulfosporosinus* and *Acididesulfobacillus*.

Focusing on the amendment of organic compounds to the cultures, Santos and Johnson show the suitability of alcohols of small molecular weight, such as methanol, ethanol and glycerol, as electron donors for biosulfidogenic processes at low pH, revealing an increase in sulfate reduction rates and the impacts on the dominant microbial communities in system. A remarkable abundance of *Desulfovibrio desulfuricans* and *Desulfosporosinus acididurans* is reported in the conditions of interest and a recommendation for crude material as electron donors is recommended. While the application of crude potential feed material appears to be a cost-effective alternative, availability and toxicity due to impurities remain to be researched.

One manuscript focuses on integrating the kinetic modelling based on low- and high-cost electron donors and the microbial profiling, providing insights to design and operation of biosulfidogenic reactors. In this study, Hessler et al. demonstrate how acetate can be used effectively despite low associated growth rates of the microorganisms, and how lactate can be used to achieve high sulfate reduction rates. Applying the most suitable parameters, high sulfate reduction rates and enrichment of microorganisms of interest can be achieved.

Two manuscripts in this *Research Topic* focus on the optimization of operational parameters in bioreactors for sulfate removal and elemental sulfur recovery. In those cases, the produced sulfide is driven to sulfide oxidation and elemental sulfur recovery from the system. Schwarz et al. report improved two-step membrane biofilter reactor systems for reduction of sulfate and oxidation of sulfide. In this study, pH is a key parameter to avoid toxicity of H_2_S in acidic conditions, but also to avoid CaCO_3_ scaling in alkaliphilic conditions. Marais et al. show the suitability of a hybrid linear flow channel reactor for simultaneous sulfate reduction and partial oxidation of sulfide, allowing elemental sulfur recovery. In this study, temperature is a key operational parameter, affecting sulfate-reducing bacteria performance and, consequently, the microbial community dynamics. Periods of low temperature could result in a more robust and efficient microbial community as temperatures transition from low to high, and this would have positive implications in real-world applications.

Overall, the studies gathered in this *Research Topic* issue illustrate how this particular area of Biotechnology is continuing to provide insights into the microbial physiology and engineering strategies to optimize biosulfidogenic processes. Overall, we hope the manuscripts here collected will provide novel, compelling and useful traits on this thrilling field of study with plenty of opportunities for further research and application.

